# Additive Manufacturing of Wood Flour/PHA Composites Using Micro-Screw Extrusion: Effect of Device and Process Parameters on Performance

**DOI:** 10.3390/polym13071107

**Published:** 2021-03-31

**Authors:** Jing Tian, Run Zhang, Jiayuan Yang, Weimin Chou, Ping Xue, Yun Ding

**Affiliations:** Department of Mechanical and Electrical Engineering, Beijing University of Chemical Technology, Beijing 100029, China; tianjing_buct@163.com (J.T.); zhangrun_buct@163.com (R.Z.); yangjy370@163.com (J.Y.); cwm758452@163.com (W.C.); dingyun@mail.buct.edu.cn (Y.D.)

**Keywords:** additive manufacturing, micro-screw extrusion, performance, printing parameter, bio-based composite

## Abstract

Based on additive manufacturing of wood flour and polyhydroxyalkanoates composites using micro-screw extrusion, device and process parameters were evaluated to achieve a reliable printing. The results show that the anisotropy of samples printed by micro-screw extrusion is less obvious than that of filament extrusion fused deposition modeling. The type of micro-screw, printing speed, layer thickness, and nozzle diameter have significant effects on the performance of printed samples. The linear relationship between the influencing parameters and the screw speed is established, therefore, the performance of printed products can be controlled by the extrusion flow rate related to screw speed.

## 1. Introduction

Additive manufacturing (AM) has been widely used in engineering and industry fields in recent years owing to processing of complex parts and customization, such as aircraft, dental restorations, medical implants, and automotive products [1,2,3]. Among many kinds of AM technologies, fused deposition modeling (FDM) technology is still expanding the types of materials owing to flexibility in design and low cost, and a lot of research has been done on FDM technology. A large number of FDM parameters have different effects on product performance [4]. However, few researches have done on AM of bio-based composites. Previous study has proposed to use micro-screw extrusion AM to resolve the discontinuous feeding and nozzle clogging of wood flour/polyhydroxyalkanoates (WF/PHA) composites without additives, and AM of WF/PHA composites has been preliminary studied [5]. Although the forming process of filament extrusion FDM has been extensively studied [6,7,8,9], few systematic studies concerning the micro-screw extrusion FDM has been published yet.

**Figure 1:**
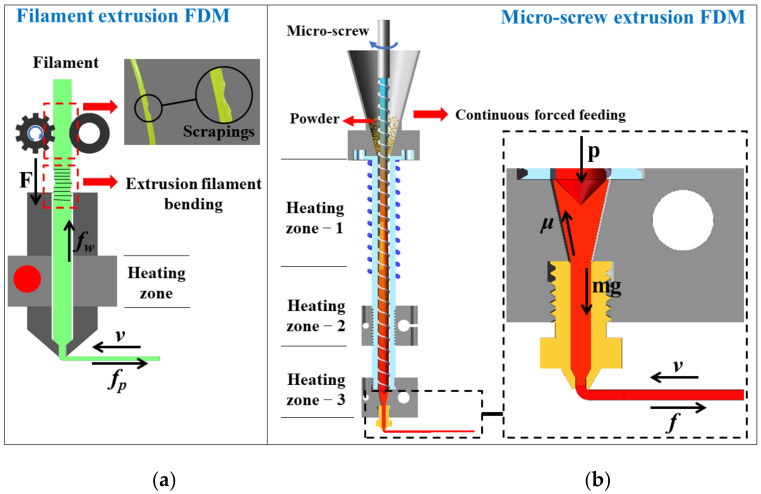
Schematic diagram of (**a**) filament extrusion and (**b**) micro-screw extrusion FDM.

The difference of FDM based on filament extrusion and micro-screw extrusion is analyzed to elaborate the importance of forming process. Figure 1 shows the schematic diagram of FDM using micro-screw extrusion and filament extrusion in the forming process. The difference is described by feeding, heating, pressure along the extrusion direction and deposition effect.

In feeding part, filament extrusion requires a specific filament diameter (1.75 or 3 mm), which restricts the diameter of the nozzle. Secondly, only a pair of gears is used to convey the filament, and the unmelted filament is used to push the molten filament to extrude. This may cause the filament to produce scrapings, break, and even slip in the fast feeding. For flexible materials, the gear clamping cannot push the molten filament and may cause the filament to bend [10], which results in failure of printing. In contrast, the feeding method of micro-screw extrusion is continuous forced feeding without nozzle clogging [11]. In addition, the allowed layer thickness of micro-screw extrusion is much higher than that of filament extrusion, which has been verified in Section 3.2.

For the heating system, the filament extrusion has only one heating zone, the filament may not keep molten during high-speed printing, which results in poor print quality. By contrast, the materials can be completely melted through three heating zones during micro-screw extrusion, thereby improving the forming quality [12,13]. Therefore, higher melting efficiency can achieve higher printing speed compared with filament extrusion FDM. 

Micro-screw extrusion gradually obtained a stable pressure along the extrusion direction [14], while filament extrusion is a kind of pressure-less extrusion.

For the deposition effect, filament extrusion deposition will produce a lot of voids between the filament [6], which affects the quality of products, while the micro-screw extrusion deposition can reduce the void by adjusting the screw speed and printing parameters [15].

Based on the above differences, it is necessary to conduct further study on WF/PHA micro-screw extrusion AM to characterize the influence of various parameters.

The main influencing parameters of filament extrusion FDM are raster angle, layer thickness, nozzle diameter, feed rate, infill density, and printing speed, etc. The significance of several process parameter is obtained through the literature evaluation of filament extrusion FDM: raster angle > layer thickness > printing speed > nozzle temperature [1,6,7,8,16,17,18,19,20,21,22,23,24,25]. 

In this study, the forming parameters of micro-screw extrusion are divided into device and process parameters. The effects of device parameters including screw type and nozzle diameter and process parameters including raster angle, layer thickness, printing speed, and nozzle temperature on mechanical and thermal properties are evaluated. For this purpose, one-way analysis of variance (ANOVA) is used to assess the effects of various parameters on micro-screw extrusion AM, and the tensile strength is used as the evaluation index. The paper is arranged as follows. Firstly, the experimental methodology carried out in this study is briefly summarized with emphasis on specimen preparation, extrusion AM parameters and the experimental set-up. Then, the effect of the device and process parameters on printed products are summarized. Finally, conclusions of this work are outlined.

## 2. Materials and Methods

### 2.1. Specimen Preparation

**Figure 2:**
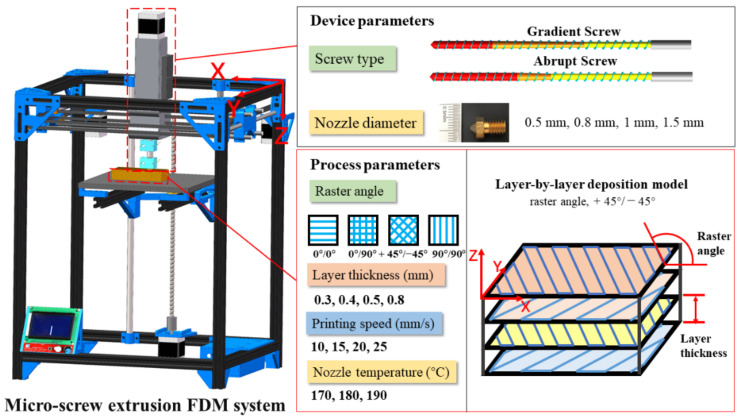
Micro-screw extrusion FDM system and forming parameters.

The WF/PHA composite with WF content of 30 wt% used in this study were prepared by a co-rotating twin-screw extruder (KS-20, Kunshan Keshun, Rubber and Plastic Machinery Co., Ltd., Kunshan, China) with a length and diameter ratio of 40. The specific composites preparation has been described in detail in previous studies [5]. WF/PHA composite samples were manufactured using the micro-screw extrusion FDM system [5] as shown in Figure 2. The standard specimens were modelled using SolidWorks software and exported as STL (Standard Triangle Language) file. The slice software was used to set process parameters and generate G-code file.

### 2.2. Extrusion AM Parameters

Based on the parameters of previous work listed in Table 1, one-way ANOVA is used to evaluate the effect of each parameter on micro-screw extrusion AM. The study on micro-extrusion AM is carried out from device and process parameters. Figure 2 shows the detail of device and process parameters used in this study. The two different screw structures and four nozzle diameters were assessed in device parameters. In total, four different raster angles, printing speed and layer thickness, and three nozzle temperature were considered in process parameters.

### 2.3. Testing and Characterization

Extrusion flow rate stability testing. The quality of the material extruded through the micro-screw extruder was tested at various temperatures and micro-screw speeds within 1 min, and each kind of sample was measured five times and the average value was taken.

Melt pressure measurement of the micro-screw extruder. The melt pressure was measured by the SEN-PT series of a mini high temperature melt pressure sensor (Wuxi Cienno Measurement and Control Technology Co., Ltd., Wuxi, China) at various temperatures and micro-screw speeds. It should be noted that ordinary filament FDM-3D printers cannot be equipped with a melt pressure sensor, while the micro-screw extrusion system designed in this study was equipped with a customized heating block for testing [5]. The micro-screw extruder was controlled to continuously extrude materials, and the data were recorded until the melt pressure value was stable.

Mechanical property testing. The micro-screw extrusion 3D printer was used to print various standard samples. The mechanical properties of samples were tested according to standard testing methods. Specifically, the tensile tests and flexural tests were conducted on a universal tester (KXWW, Chengde Taiding Testing Machine Manufacturing Co., Ltd., Chengde, China) with a load cell of 5 kN. The tensile strengths of samples were measured according to GB/T 1040.2–2006 with a stretching rate of 10 mm/min. The three-point flexural tests were conducted according to GB/T 9341–2008 with a cross-head speed of 10 mm/min. Unnotched impact strength tests were conducted on an impact tester (KBANM-II, Chengde Taiding Testing Machine Manufacturing Co., Ltd., China) according to GB/T 1843–2008. For each data point, at least 5 to 10 samples were tested.

Heat deflection temperature (HDT). Heat deflection temperature was tested by an HDT test equipment (KXRW-300CL-3, Chengde Taiding Testing Machine Manufacturing Co., Ltd., China). Based on the test principle of three-point simply supported beam structure, the printed standard samples (80 × 10 × 4 mm^3^) were tested according to GB/T1634–2004. The span of 64 mm, the load of 0.45 MPa, and the heating rate of 120 °C/h is used in HDT test procedure; in addition, the heat transfer medium is methyl silicone oil. Each datum is the average value obtained by testing 3 samples.

Scanning electron microscopy (SEM). The morphology of the impact fracture surfaces of the composites was observed by a SEM at room temperature. A SEM of Crossbeam 550 (Zeiss, Germany) with a field emission gun and accelerating voltage of 10 kV was used to collect SEM images for the composite specimens. A gold coating was placed on the impact fracture surfaces to improve the conductivity of the specimen, and the samples were viewed perpendicularly to the fractured surface. 

Density testing. A multifunctional density tester (AR-300VP, Hongtuo Instrument Co., Ltd. Dongguan, China) was used to obtain the apparent density of the sample at a room temperature. During the test, the mass *m*_1_ of the sample was tested first in the air, and then the sample was suspended in water to measure its mass *m*_2_. The apparent density (*ρ*) of the sample was calculated according to the Equation (1), and the test was repeated 5 times to get the average value.

(1) $$\rho =\frac{m_{1}\rho_{w}}{m_{1}-m_{2}}$$

where *ρ* is the apparent density of the sample (g/cm^3^), *m*_1_ is the mass of the sample in the air (g), *m*_2_ is the mass of the sample suspended in water (g), *ρ_w_* is the density of water, and its value is 1 g/cm^3^.

## 3. Results and Discussion

**Figure 3:**
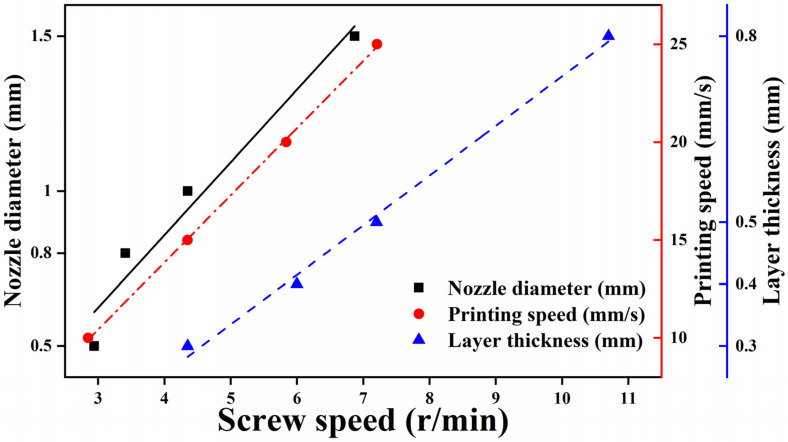
Relationship between screw speed and forming parameters.

Since the forming parameters based on micro-screw AM directly or indirectly affect the performance by extrusion flow rate, the printing parameters can be determined by establishing the relationship between forming parameters and extrusion flow rate. A previous study [5] has shown that the effect of screw speed on extrusion flow rate is more significant than that of temperature. Therefore, the relationship between screw speed and forming parameters is shown in Figure 3. The screw speed (S) is highly linear with the layer thickness (L), printing speed (V_p_), and nozzle diameter (D_N_), respectively, and the linear relationship is obtained by linear fitting, L = 0.08S − 0.0653 with goodness of fit (R^2^) of 0.9923, V_p_ = 3.43S + 0.135 with R^2^ = 0.99932, and D_N_ = 0.235S − 0.08 with R^2^ = 0.93495. In this way, the forming parameters can be determined by the relationship between extrusion flow rate and screw speed. In addition, it can be seen from the Figure 3 that the layer thickness has the most significant impact on the screw speed.

### 3.1. Effect of Device Parameters on the Micro-Screw Extrusion AM

**Figure 4:**
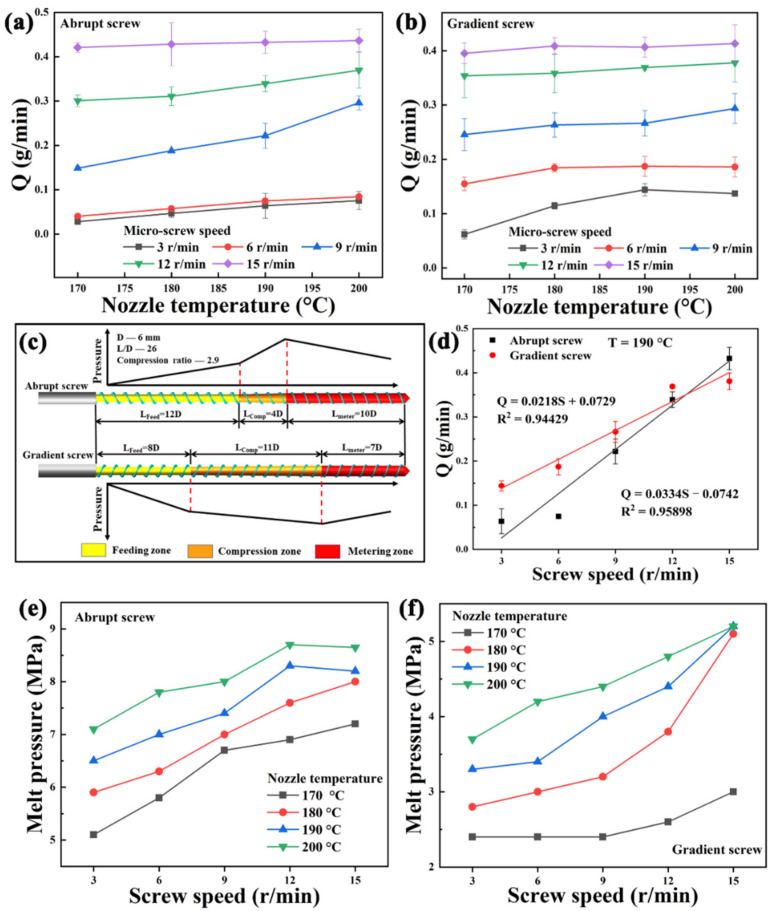
Extrusion stability curves of (**a**) abrupt screw and (**b**) gradient screw extruded at various nozzle temperatures and screw speeds. (**c**) Schematic diagram of pressure gradient curves and the structural parameters of two micro-screws. (**d**) Q-S curves of two kinds of screws at the nozzle temperature of 190 °C. Melt pressure curves of (**e**) abrupt screw and (**f**) gradient screw at various nozzle temperatures and screw speeds.

The structures of two micro-screws are shown in Figure 4c. The device parameters mainly affect the AM by the extrusion flow rate, so the extrusion flow rate stability of two screws was tested. It is necessary for polymer to be compressed during the extrusion process. First, it can increase the melting rate. Secondly, the gas between the particles can be discharged from the hopper, otherwise, the product will exhibit many defects due to voids generated inside. Finally, the higher system pressure ensures the compactness of the product. Therefore, the pressure build-up capabilities of two screws were evaluated.

#### 3.1.1. Effects of Screw Structure on Extrusion Stability and Melt Pressure

The two micro-screws exhibit many differences in the length of feeding section, compression section and metering section, and the extrusion flow rate (Q) depends on metering section. The Q of two micro-screws at various nozzle temperatures and screw speeds is shown in Figure 4a,b. Firstly, it can be concluded that screw speed is more sensitive to Q than that of temperature. Then, the Q of gradient screw is higher than that of abrupt screw when the screw speed is below 13 r/min. This is because the metering section of gradient screw is shorter than that of abrupt screw, and the efficiency of conveying the melt is much higher. Moreover, the Q of the two screws tends to be the same when the screw speed is high enough. 

Figure 4d shows Q-S curves of two kinds of screws at the nozzle temperature of 190 °C. The Q-S curves of two screws are basically linear, and the linear correlation equation is obtained by linear fitting, respectively, Q_A_ = 0.0334S – 0.0742 with R^2^ = 0.95898 and Q_G_ = 0.0218S + 0.0729 with R^2^ = 0.94429. The Q-S curve slope of the abrupt screw is higher than that of gradient screw, therefore, the Q response of the abrupt screw is better than that of gradient screw.

The reason of the pressure growth for the micro-screw extrusion is that the groove depth of the screw gradually becomes shallow, and the resistance comes from the nozzle. The peak pressure appears at the front of the metering section or the back of the compression section. As shown in Figure 4c, the compression section of the gradient screw starts earlier than that of abrupt screw and therefore the pressure of the former is built in advance. Based on this reason, the conveying capacity and melting efficiency of the gradient screw are higher than those of abrupt screw, and the different Q are obtained. 

Although the melt-conveying efficiency of the abrupt screw is lower than that of the gradient screw, the pressure build-up ability of the former is better. The melt pressure curves of two micro-screws at various nozzle temperatures and screw speeds are shown in Figure 4e,f. The melt pressure of gradient screw has no significant change at lower screw speed (<9 r/min) and temperature. The trend of melt pressure with screw speed is consistent with Q-S curves. The melt pressure increases with screw speed and temperature, but the effect of temperature is more significant. Basically, the reason is that the rotation of the screw can make the material extrude from the nozzle, but the change of temperature cannot. The fluidity of the material increases with the temperature, and the material accumulates in the melt chamber under the action of gravity, thus increasing the melt pressure. In addition, compared with Figure 4a,b, the Q value of gradient screw is larger than that of abrupt screw, so the melt pressure of abrupt screw is higher than that of gradient screw.

#### 3.1.2. Effects of Screw Structure on Mechanical and Thermal Properties

**Figure 5:**
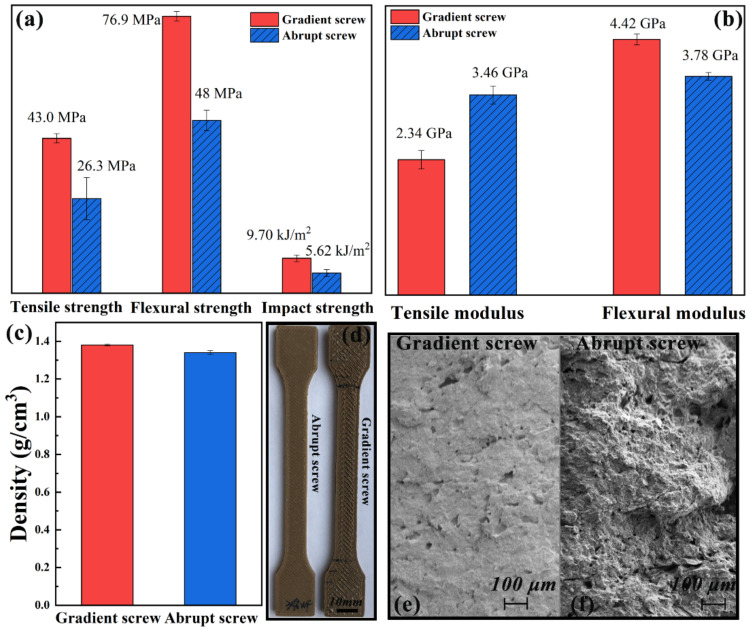
(**a**) Mechanical strength and (**b**) modulus, (**c**) density, (**d**) surface quality and (**e**,**f**) SEM images of samples printed by two kinds of screws.

The mechanical strength and modulus of samples printed by two micro-screws at the same printing parameters are shown in Figure 5a,b. It can be obviously seen that the mechanical strength of gradient screw is much higher than that of abrupt screw. This is because the corresponding screw speed is 4–5 r/min when the layer thickness is 0.3 mm, and the Q of gradient screw is greater than that of abrupt screw, so the deposition of gradient screw is denser at the same deposition rate. This can be further verified by the density, surface deposition, and SEM images of the fractured cross-section of samples in Figure 5c–f. It can be seen from Figure 5d that the surface of the sample printed by gradient screw is uneven due to excessive extrusion compared with that of abrupt screw. SEM images show that the sample printed by abrupt screw has more voids compared with that of gradient screw. This is consistent with the density of samples printed by two kinds of screws.

**Figure 6:**
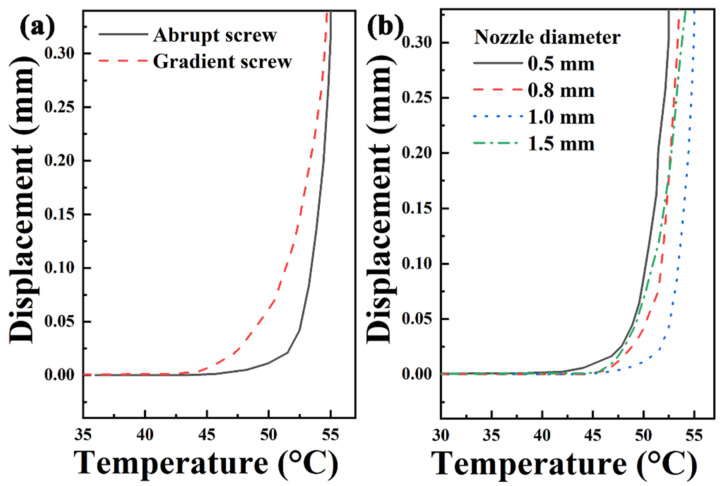
HDT curves of samples printed by (**a**) two kinds of screws and (**b**) various nozzle diameters.

The heat deflection resistance of samples printed by two kinds of screws is shown in Figure 6a. Although the final HDT of samples printed by two screws almost the same, the deformation initiation temperature of sample printed by gradient screw is lower than that of abrupt screw. This is because the deposition of gradient screw is more than that of abrupt screw, and the internal stress increases, which leads to the advance of the deformation initiation temperature.

#### 3.1.3. Effects of Nozzle Diameter on Mechanical and Thermal Properties

**Figure 7:**
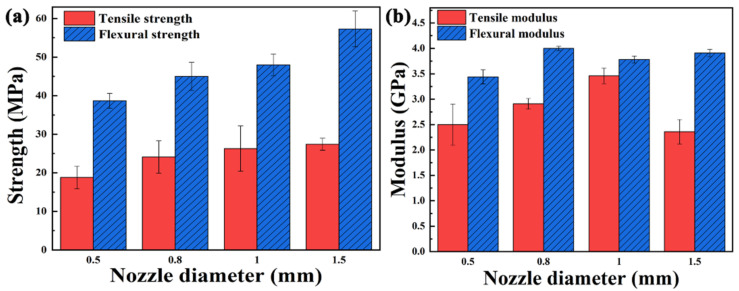
(**a**) Mechanical strength and (**b**) modulus of samples printed by various nozzle diameters.

The nozzle diameter directly affects the extrusion flow rate by extrusion pulse [5]. The mechanical strength and modulus of samples printed by various nozzle diameters are shown in Figure 7. It can be seen that mechanical properties increase with the nozzle diameter. The smaller the nozzle diameter is, the better the surface quality is. However, the smaller nozzle diameter means more printing paths, resulting in more interface and lower strength of the printed sample. Figure 6b shows the HDT of samples printed by various nozzle diameters. HDT increases with the nozzle diameter. HDT is directly proportional to the modulus and inversely proportional to the applied load at which deformation occurs [26]. Therefore, the decreased modulus of sample printed by 1.5 mm nozzle diameter was also a factor in the decrease in HDT.

### 3.2. Effect of Process Parameters on the Micro-Screw Extrusion AM

#### 3.2.1. Mechanical Properties

**Figure 8:**
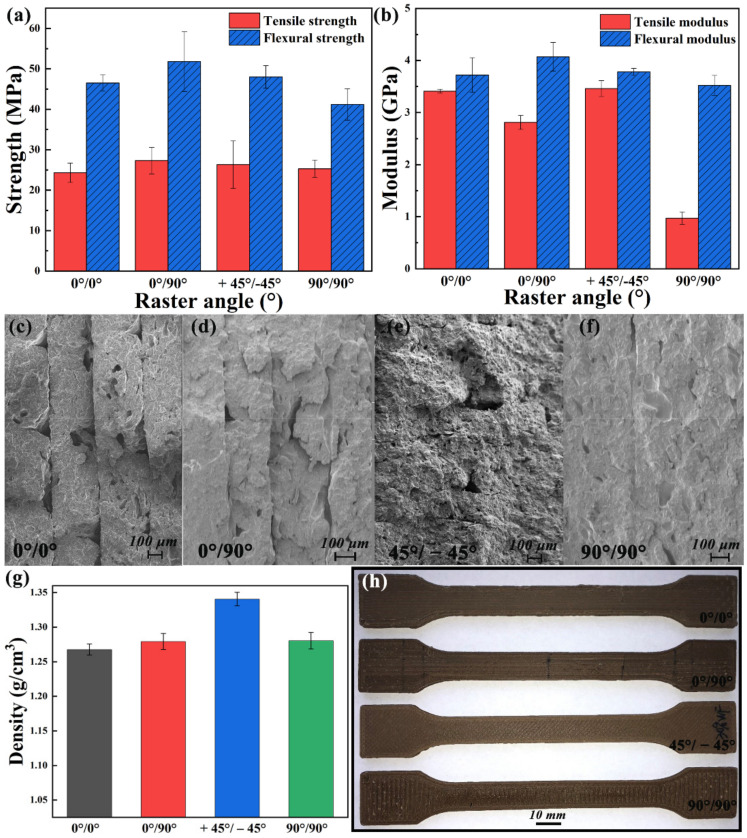
(**a**) Mechanical strength and (**b**) modulus, (**c**–**f**) SEM images of the fractured cross-section, (**g**) density and (**h**) surface quality of samples printed at various raster angles.

Raster angle. The properties of abrupt screw extrusion 3D printed samples with various raster angles are shown in Figure 8. The mechanical properties of samples deposited in the single direction (0°/0°, 90°/90°) are lower than those of others from Figure 8a,b.The mechanical properties of the sample printed with 0°/90° raster angle are the best. In the tensile test, the specimen can bear more loads when the printing paths are parallel to the loading direction [27]; therefore, the tensile modulus is the highest at 0°/0°raster angle. It is noticed that the tensile modulus of 90°/90° raster angle drops sharply because the direction of tensile force is perpendicular to the deposition direction, which can be used to indirectly characterize the adhesion between layers.

SEM images of the fractured cross-section of samples printed at various raster angles are shown in Figure 8c–f. The interface between layers can be observed in samples deposited at 0°/0° and 0°/90° raster angle. The 45°/−45° raster angle has a tighter stacking than that of 0°/0°, 0°/90°, and 90°/90°, which reflected in the increased density (Figure 8g) and reduced interface on SEM images. The influence of raster angle on the surface quality of samples cannot be ignored. As shown in Figure 8h, the surface of 45°/−45° raster angle is the most flat compared with the other three raster angles. However, in conclusion, the effect of raster angle on the tensile strength of samples is not significant (24.3–27.3 MPa).

**Figure 9:**
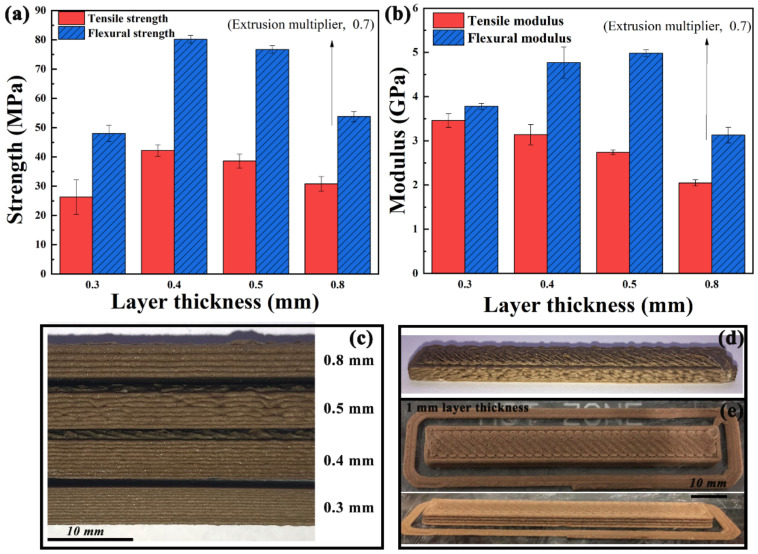
(**a**) Mechanical strength and (**b**) modulus, and (**c**) morphology in layer thickness direction of samples printed with various layer thicknesses. (**d**) Over-extrusion in 0.8 mm layer thickness. (**e**) 1 mm layer thickness test print.

Layer thickness. Layer thickness has a wide range of adjustable screw speed (4–11 r/min) from Figure 3. Therefore, the effect of layer thickness on the properties of samples is crucial. The mechanical properties of samples printed with various layer thicknesses are shown in Figure 9a,b. Compared with filament extrusion, micro-screw extrusion FDM does not have better mechanical properties with lower layer thickness as reported in [27,28,29,30], but there is an optimal layer thickness to obtain the highest performance. The best mechanical strength can be obtained when the thickness is 0.4 mm. The tensile strength and flexural strength can reach 42.2 and 80.2 MPa, respectively. The morphology in layer thickness direction of samples is shown in Figure 9c. It can be seen that the smaller the layer thickness is, the better the surface quality is [6,31], but the increase in the interface leads to the decrease in the strength. However, it is not the less the interface is, the higher the strength is, but the mechanical properties begin to decrease when the layer thickness is higher than 0.4 mm. This is because the higher the layer thickness is, the higher the screw speed is, and the extrusion flow rate does not match the screw conveying amount. Then, the material accumulates in the nozzle melt chamber, which may produce degradation and affect the performance of deposited samples.

Notably, when the layer thickness is 0.8 mm, the extrusion multiplier is reduced from 0.9 to 0.7 to complete the sample printing due to serious over-extrusion (Figure 9d). Even if the extrusion multiplier is reduced by 0.2, the screw speed corresponding to 0.8 mm layer thickness still can reach 11 r/min. In other words, it is verified that 1 mm nozzle diameter can print 0.8 mm layer thickness or even thicker. In addition, the sample printed with 1 mm layer thickness was tested, as shown in Figure 9e. 

It can be concluded that the micro-screw extrusion AM is not limited to the nozzle diameter [6], allowing higher layer thickness than that of filament FDM. The higher layer thickness can be printed by controlling the screw speed to provide sufficient extrusion output. 

**Figure 10:**
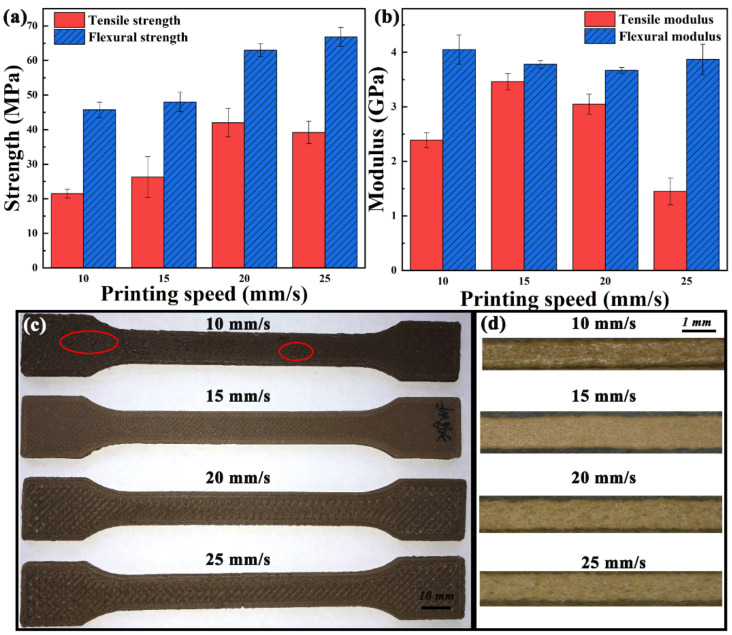
(**a**) Mechanical strength and (**b**) modulus, and (**c**) surface quality of samples printed at various printing speeds. (**d**) Microscope images of deposited filament morphology at various printing speeds.

Printing speed. The effect of printing speed on the mechanical properties are shown in Figure 10a,b. The mechanical strength of the printed sample increases with the printing speed. Fast printing speed ensures that the previous layer is not completely cooled, and then deposits the next layer, thereby improving the bonding of interlayers. However, further increase in printing speed will result in decreased mechanical properties [19].

Figure 10c shows the surface quality of samples printed at various printing speeds. Combined with the relationship between printing speed and screw speed in Figure 3 and Q-S curve of abrupt screw in Figure 4d, slow extrusion rate leads to insufficient filling when the printing speed is 10 mm/s. The extrusion flow rate increases with the printing speed. The surface of the sample is the flattest at the printing speed of 15 mm/s, and obvious printing path appears on the surface of the sample printed at 20 mm/s. This is owing to the mismatch between the extrusion flow rate and filament width. Figure 10d shows the morphology of extruded and deposited single filament. With the increase in printing speed, the color of the filament becomes shallow, which is because the residence time of the material in the nozzle melt chamber is reduced. When the printing speed increases, it means that the screw speed increases, and the extrusion rate from the nozzle increases, which may lead to the generation of voids in filaments and narrowing of the filament due to unstable extrusion [32].

**Figure 11:**
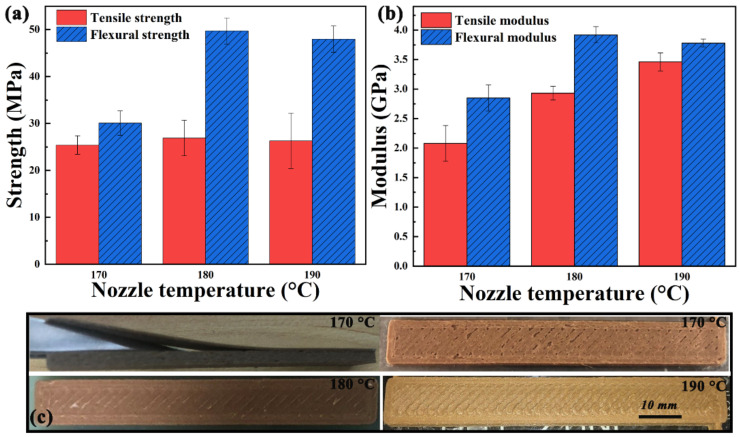
(**a**) Mechanical strength and (**b**) modulus, and (**c**) surface quality of samples printed at various nozzle temperatures.

Nozzle temperature. Although the temperature is a key parameter affecting the material properties, it can be seen from Figure 11a that the nozzle temperature has little effect on the tensile strength of printed sample. The effect of temperature on mechanical properties is consistent with that of extrusion flow rate. The effect of melting temperature (170–190 °C) on flexural strength is significant, which is due to low extrusion flow rate and insufficient filling at 170 °C (Figure 11c). It can be seen from Figure 11b that the modulus increases with the nozzle temperature, which is because the bonding force of interlayers increases with the nozzle temperature. Figure 11c shows the surface quality of samples printed at various nozzle temperature. The layers are even separated due to the poor interlayer bonding [33] when the nozzle temperature is 170 °C. 

#### 3.2.2. Thermal Property

**Figure 12:**
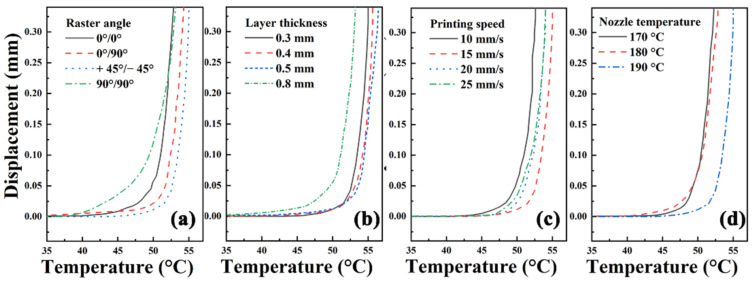
HDT curves of samples printed at various (**a**) rater angles, (**b**) layer thicknesses, (**c**) printing speeds and (**d**) nozzle temperatures.

HDT of crystalline polymers is sensitive to thermal history [34]. The AM process of stacking layers is also the process of internal stress accumulation, so the HDT of printed samples may be different from that of traditional manufactured samples. The effect of process parameters on the thermal property of printed samples is characterized by HDT. Figure 12 shows the HDT curves of samples printed at various process parameters. The raster angle and nozzle temperature have significant effects on HDT of printed samples. The thermal deformation resistance of samples deposited in single direction is poorer than that in cross direction as shown in Figure 12a. It can be seen from Figure 12b that the thicker the layer is, the stronger the ability to resist thermal deformation. As shown in Figure 12c, HDT reaches the highest when the printing speed is 15 mm/s. Figure 12d shows that HDT increases with the nozzle temperature. The interlayer bonding force increases with the nozzle temperature; therefore, the ability to resist deformation is stronger.

### 3.3. Effect Assessment of Device and Process Parameters on the Micro-Screw Extrusion AM

One-way ANOVA is used to evaluate the effect of various parameters on the micro-screw extrusion AM, and the tensile strength is used as the evaluation index. Figure 12a shows the trend of tensile strength with each parameter. The data from One-way ANOVA are listed in Table 2. Within groups sum of squares, also known as residual sum of squares, reflects the difference between the sample value and the sample mean at a certain level. Between groups sum of squares, reflects the difference between the sample mean and the total mean of the sample at each level, which is caused by different levels of factors. The significance of each parameter on tensile strength is determined by comparing the between groups sum of squares relative to within groups sum of squares. It can be seen intuitively that the layer thickness, printing speed and screw type have a significant effect on the tensile strength. 

The significance of each parameter can be obtained from the data in Table 2: Printing speed > Layer thickness > Screw type > Nozzle diameter > Rater angle > Nozzle temperature.

Printing time of a tensile sample was further evaluated to characterize the printing efficiency. The printing time of raster angle, nozzle temperature and screw type are the same (50 min). 

**Figure 13:**
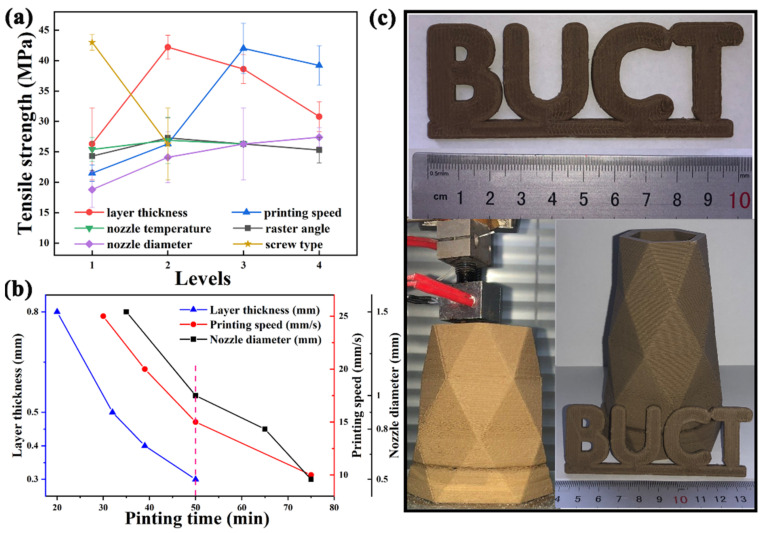
(**a**) Tensile strength curves and (**b**) Printing time curves of samples printed at various effect parameters. (**c**) Printed products.

The curves of printing time at various layer thickness, printing speed and nozzle diameter of samples are shown in Figure 13b. Compared with Figure 3 and Figure 13b, the higher the screw speed is, the shorter the printing time is. Therefore, larger nozzle diameter, higher layer thickness, and printing speed are conducive to reducing printing time.

Overall, choosing the appropriate micro-screw extrusion AM parameters can obtain better product performance. Figure 13c shows the product printed with the optimal parameters, which has few defects of insufficient filling or excessive extrusion.

## 4. Conclusions

In this study, the forming parameters affecting the micro-screw extrusion AM include device parameters of the screw type and nozzle diameter, and process parameters of the rater angle, layer thickness, printing speed, and nozzle temperature. These parameters are directly or indirectly related to the screw speed. The linear relationship between screw speed and forming parameters was established. The mechanical and thermal properties of various parameters for micro-screw extrusion AM were evaluated, and the differences between filament extrusion and micro-screw extrusion FDM were compared and summarized as follows:

(1) The anisotropy of micro-screw extrusion FDM was not as obvious as that of filament extrusion, which reflected in raster angle;
(2) The printing speed is the most significant factor for micro-screw extrusion AM, followed by the layer thickness. Therefore, the printing speed and layer thickness should be primarily considered in the printing parameters of micro-screw extrusion FDM to obtain better surface quality and performance;
(3) The structure of two kinds of micro-screw has different effect on the extrusion flow rate and melt pressure, resulting in the mechanical properties of samples printed by gradient screw are significantly higher than that of abrupt screw;
(4) Although the increase in printing path can improve the surface quality, it will increase more interfaces between the melted filaments (interlayers) and reduce the mechanical properties;
(5) Compared with filament extrusion FDM, higher thickness can be obtained by micro-screw extrusion. The excellent mechanical properties (tensile strength and flexural strength) were obtained with layer thickness of 0.4 mm, while the better surface quality was obtained with layer thickness of 0.3 mm. Therefore, the layer thickness should be weighed between strength and surface quality;
(6) The effect of nozzle temperature on tensile strength is not significant compared with flexural strength. Specifically, the printed sample even appears delamination of deposition layers at low temperature in flexural test, however, it will lead to the degradation of WF/PHA bio-based composite at too high temperature and affecting the performance of products;
(7) The influence of nozzle temperature on thermal performance is more significant than that of other parameters due to internal stress;
(8) Micro-screw extrusion FDM can control the mechanical properties of products by the extrusion flow rate.

In conclusion, the effects of device and process parameters on mechanical and thermal properties of samples printed using micro-screw extrusion were comprehensively studied, which provided conditions for the AM of bio-based composites. The relationship of process-structure-property of micro-screw extrusion AM was further clarified.

## Figures and Tables

**Table 1:** Device and process parameters in the previous work.

Screw Type	Nozzle Diameter	Raster Angle	Layer Thickness	Printing Speed	Nozzle Temperature	Extrusion Multiplier ^a^
Abrupt screw	1 mm	+45°/−45°	0.3 mm	15 mm/s	190 °C	0.9

^a^ Extrusion multiplier is the ratio between actual and theoretical extrusion quantity.

**Table 2:** Data from one-way ANOVA.

Parameters	Within Groups Sum of Squares	Between Groups Sum of Squares
Raster angle	60.6	17.3
Layer thickness	55.6	491
Printing speed	70.4	944
Nozzle temperature	63.0	4.54
Nozzle diameter	80.1	173
Screw type	11.8	349

## Data Availability

Not applicable.
